# Athletic identity affects prevalence and disclosure of emotional abuse in Finnish athletes

**DOI:** 10.3389/fspor.2024.1406949

**Published:** 2024-06-05

**Authors:** Jatta Muhonen, Ashley Stirling, Marja Kokkonen

**Affiliations:** ^1^ Institute of Criminology and Legal Policy, University of Helsinki, Helsinki, Finland; ^2^ Faculty of Kinesiology & Physical Education, University of Toronto, Toronto, ON, Canada; ^3^ Faculty of Sport and Health Sciences, University of Jyväskylä, Jyväskylä, Finland

**Keywords:** emotional abuse, psychological abuse, interpersonal violence, athletic identity, disclosure

## Abstract

The present study offers novel insight into the topic of experienced and observed emotional abuse by researching factors that affect athletes' responses to emotional abuse by coaches. The research aimed to explore three main questions: (1) whether athletic identity was associated with the prevalence of emotionally abusive coaching practices, and (2) disclosure of emotional abuse, and (3) whether demographic variations existed in the frequency of emotional abuse, athletic identity, and disclosure of the abuse. Study participants who filled in an anonymous digital survey consisted of athletes from elite to leisure levels living in Finland (*N* = 3687, aged 12–80, gender 61% female, 37.7% male, 0.8% other genders). The research findings highlighted three key insights. Firstly, Pearson correlations revealed that a salient athletic identity was related to a higher prevalence of emotional abuse. Secondly, ANOVA/Kruskal-Wallis tests between-groups indicated that particularly children were susceptible to the abuse. Thirdly, a mediation analysis showed that self-identity (aspect of athletic identity) influenced the relationship between experienced emotional abuse and disclosure, by reducing disclosure. As a result, holistic identity development is recommended for athletes and particularly children in sports.

## 1 Introduction

Over the past few years, several high-profile cases of emotional abuse have emerged in sports environments worldwide. One of these is that of a Finnish synchronized skating coach who, according to reports, repeatedly and intentionally emotionally abused their athletes for several years by, for instance, telling their athletes, including children, to kill themselves (1). The case was considered a landmark case by many industry professionals. It highlighted that the issue of violence by coaches, and that of emotional abuse is very much present in Finnish sports (1). Similar cases can be observed in sports environments worldwide. For instance, in the UK, Olympians Becky and Ellie Downie revealed in 2020 experiencing years of emotional abuse in British Gymnastics (2). In Canada, stories continuously emerge about abuse in ice hockey (3). Thus, demonstrating that abuse in sports is not constrained to any one culture or region; it is a global concern that requires immediate attention (4).

Emotional abuse is a form of violence described as “a pattern of deliberate non-contact behaviors by a person within a critical relationship role that has the potential to be harmful” by Stirling and Kerr (5; p. 178). It is a form of relational violence characterized by an abuse of power within a critical relationship (6). Violence, used here interchangeably with abuse, stands for various activities that may be harmful to athletes, including maltreatment, neglect and exploitation (7). Emotional abuse can manifest itself through verbal behaviors (e.g., shouting, humiliating and threatening), denial of attention and support (e.g., exclusion from training or lack of feedback) and physical (non-contact) behaviors (e.g., throwing of objects or punching a wall). According to various prevalence studies, emotional abuse (or psychological abuse) is the most common form of violence in sports (4, 8) with its prevalence being around 38%–75% in various disciplines [e.g., (9, 10)]. For instance, in a study on vulnerable group of Finnish gender and sexual minority sport participants, 27%–35% reported sexual or gender-based verbal or non-verbal harassment by a coach (11), and 33%–50% sexual or gender-based harassment and 4%–19% physical abuse by fellow sport participants in teams or groups (12).

### 1.1 Coach as the perpetrator

While the perpetrator of emotional abuse can be an athlete, coach, mentor, director of a sport club or parent (13, 14), in the present article we focus on the coach as the perpetrator and the athlete as the survivor of the abuse. The distinction was made because coaches alongside peers are the most common perpetrators of emotional abuse, and due to the unique position coaches hold in relation to athletes (15). Emotional abuse in the athlete-coach relationship is categorized as relational abuse, as it exists within a significant relationship that is characterized by the unequal status of coach and athlete (5). Coaches have considerable influence over athletes due to their age, knowledge, and control over the athlete's career progression (16, 17). Athletes, especially children, rely on their coaches for guidance and expertise which are vital for their development and success (17, 18). This dependence can lead young athletes to view their coaches as infallible authorities, leaving them susceptible to emotional abuse (19). In line, emotional abuse by coaches is documented to have severe consequences on athletes' wellbeing (20, 21). These encompass a broad range of emotional, social, physical, and cognitive effects (15, 22).

### 1.2 Influence of athletic identity

As illustrated above, the majority of past studies on emotional abuse have focused on prevalence, perpetration and experiences as well as the effects of emotional abuse by coaches [e.g., (5, 10, 20)]. Recently, a growing body of research has begun to emerge on factors contributing to athletes' experiences of abuse, which may subject them to additional risks of abuse [e.g., (4, 23)]. One such factor could be athletes' athletic identity. Stirling and Kerr (24) suggested that athletic identity might affect athletes' ability to recognize and react to emotional abuse. Athletic identity is defined as the degree to which an individual identifies themselves within the athlete role and looks to others for acknowledgement of that role (25, 26). Athletes develop their athletic identities through learning the psychological and situational factors such as norms and values of a sport (26). Most athletes tend to identify themselves through the lens of their engagement in sports only and develop salient athletic identities (27). According to Stirling and Kerr (24), athletes with a salient athletic identity could have a harder time recognizing and reacting to emotionally abusive coaching practices compared to other athletes due to a process of normalization. Abusive behaviors such as shouting are generally normalized in sports to a degree, where these behaviors are perceived as acceptable (19). Athletes normalize abusive behaviors for various reasons, including success, denial, fear of consequences, and the norms and culture of a sport (28, 29). The more salient an athlete's athletic identity becomes, the more likely they are tonormalize and adhere to the norms of sport (30).

### 1.3 Norms of sports

The norms of sports, often referred to as sport ethical norms, constitute the criteria set by the sports industry to outline the characteristics of a “true athlete” (31). According to these athletes are expected to push through pain, reject limitations, embrace risks, pursue excellence, and make sacrifices. Interestingly, the norms of sport form aspects of athletes' athletic identity (25, 26). Athletes get socialized into and learn the sport norms quickly upon entering the sport context for instance through personal and behavioral observation of more seasoned athletes and especially authority figures (32–34). When more experienced athletes exhibit behaviors that endorse specific norms and practices—such as emotionally abusive coaching behaviors—younger athletes who observe this behavior learn to perceive these norms as commonplace within the environment, regardless of their potential harmfulness (34, 35). In other words, the coaches' behavior becomes normalized, and the younger athletes internalize and conform to the norms (36). This is something coaches too endorse, by rewarding adherence to the norms and punishing nonconformity (23). To athletes challenging any harmful norms within sports may, to some extent, be perceived as a threat to their career, performance and athletic identity (27, 30). Furthermore, the sport ethic norms are glorified by media, sports club officials, and fans, pushing athletes to align with these norms (23, 35). The potential danger with this process is that athletes might internalize, normalize and inadvertently accept harmful sport norms such as emotional abuse by coaches (36). It is also plausible athletes become unable to recognize abusive behaviors, exposing athletes to further abuse (32). Consequently, conformity to aspects of sport ethic norms have been shown to increase the prevalence of abuse in sports (23). The norms could also discourage athletes from disclosing instances of abuse (37).

### 1.4 Disclosure of emotional abuse

According to research, athletes generally do not tend to disclose their experiences of abuse [e.g., (9, 38)]. For instance, Kerr and colleagues (39) found that of athletes who responded to their survey, only 16% had reported their harmful experiences in sports. While the term disclosure has traditionally lacked clarity in sport psychology research, here disclosure (sometimes referred to as self-disclosure) refers to an athlete telling another person about abusive experiences in sports (40, 41). Disclosure of harm is argued to be a complex process, with numerous potential barriers (42). Despite limited research on the barriers, insights can be drawn from social psychology. Minto et al. (43) suggested that individuals with a strong group identity tend to avoid reporting abuse. When individuals view their group as positive and ethical, they are less likely to report abusive behaviors because of their strong sense of belonging and a desire not to deviate from group norms (44). Athletes, who often have a deep sense of identity tied to their teams, may be hesitant to disclose emotional abuse due to this strong association with their sports group (31). Thus, we propose that such athletic identity may act as a barrier preventing athletes from recognizing and disclosing emotionally abusive behavior by coaches (24). It is of paramount importance that athletes can disclose emotionally abusive coaching practices, as it is the primary method for detecting abusive behaviors in sports (37).

### 1.5 Research questions

Although we have outlined the various potential pathways through which athletic identity could influence the prevalence, and disclosure of emotional abuse by coaches, it is imperative to acknowledge the absence of empirical data substantiating the connection between the variables. Hence, the main aim of the present study is to ascertain the presence of any correlation between emotional abuse, athletic identity, and disclosure of abusive behaviors. To further address the limitations of past research, and to research the arguments made by Stirling and Kerr (24), our research questions were threefold: (1) is there an association between athletic identity and the prevalence of emotional abuse by coaches? (2) is there a relationship between athletic identity and disclosure of emotional abuse by coaches? and (3) are there demographic differences in the prevalence of emotional abuse, athletic identity and disclosure of the abuse? The term athletic identity here refers to the terminology by Brewer and colleagues (25), and emotional abuse to the definition of Stirling and Kerr (5). Based on past research we expected a salient athletic identity to be a factor exposing athletes to emotionally abusive coaching practices by preventing athletes from disclosing emotional abuse by coaches. It is important to acknowledge that previous relevant research has predominantly employed qualitative methodologies and concentrated on adult athletes (33). The advantage of quantitative research would be the arguably greater generalizability, reliability of research results and large range of potential analyses (Figgou & Pavlopoulos, 2015). To overcome past research limitations, our study adopted a quantitative approach and aimed to expand the participant group to include children (under the age of 18). Furthermore, previous research on emotional abuse has almost solely focused on athletes' personal experiences of emotional abuse. However, in several cases, emotionally abusive behaviors are witnessed by more than one person. Athletes' observations of emotional abuse should also be researched, first, as bystander action increases the likelihood of investigation and sanctions for a perpetrator of abuse (45). Secondly, also observed abuse appears to have detrimental ramifications and failure to take action may extend the suffering of the witnesses (16, 46). Therefore, the present study focused both on experienced and observed emotional abuse.

## 2 Materials and methods

### 2.1 Participants and their recruitment

The sample consisted of 3,687 participants living in Finland. Majority of the participants were women, secondly men and third of other genders (Table 1). The minimum participation age was 12 years old, and no maximum participation age existed (*M* = 27.91, SD = 1.18, range = 12–80). The age range was justified because children from 12 years onwards can be expected from their cognitive skills to be able to answer the research survey (47). We collaborated with a children's rights associate professor to ensure our survey was suitable for children, and we distributed the same questionnaire to both adults and children. Additionally, given the substantial variability in peak and upper age limits across various sport disciplines, this age range was deemed appropriate.

**Table 1:** Demographics of the participants.

Category	Subcategory	*n*/%
Genders	Female	2,004/54.4%
	Male	1,510/41%
	Other	23/0.6%
	Did not wish to disclose	38/1%
Age	12–17	1,029/27.9%
	18–30	1,177/31.9%
	31–40	564/15.3%
	41–60	845/21.8%
	61–80	42/1.1%
Level of proficiency	International/professional athlete	378/10.3%
	National level athlete	1,016/31.2%
	Regional level athlete	885/27.6%
	Active exerciser	448/12.2%
	Regular exerciser	570/15.5%
	Occasional exerciser	225/3.8%
	Did not wish to disclose	90/6.1%
Equity deserving groups	Deaf/deaf athlete	8/0.3%
	Para-athlete	13/0.8%
	Swedish speaking minority	103/4.5%
	Sexual minority	148/10.1%
	Gender minority	19/11.3%
	Ethnic minority	25/12.3%
	Something else	60/14.7%
	No minority	2,837/98.7%
	Did not wish to disclose	39/1.3%
Sport type	Individual	1,689/44.9%
	Team or pair	1,990/55.1%
Most represented disciplines	Finnish baseball	424/11.8%
	Floorball	281/7.6%
	Figure skating disciplines	219/6.4%
	Ice-hockey	187/5.4%
	Equestrian sports	158/4.6%

The majority of the study's participants were athletes competing at a regional or national level. Collectively, they represented a wide spectrum of athletic disciplines, encompassing 80 different sports. Overall participation from different disciplines spread relatively evenly across different sports. Popular sports in Finland such as Finnish baseball, floor ball, figure skating disciplines (especially synchronized skating) and ice hockey were the most represented. However, it is important to note that the representation within the sample was not fully diverse. Individuals from equity-deserving groups were less represented.

We used a self-selection sampling method to target athletes and leisure exercisers for our study, allowing participants to volunteer willingly. Potential participants were approached through the Finnish Olympic and Paralympic Committees, 45 Finnish sports associations and all Finnish sports academies and training centers. National organizations were contacted via email, with follow-up discussions as needed. While no organization declined to participate, 10 did not respond to inquiries. Organizations selected their own survey distribution method (i.e., newsletter, social media, email and website) and received two reminders for timely dissemination. Using REDCap, a digital, anonymous survey was sent to athletes living in Finland, across all levels in November 2021 with a 1-month response window. Prior to data collection, the research design was reviewed and approved by the University of Helsinki's ethical review board in the humanities and social and behavioral sciences. A data protection impact assessment was conducted and approved by the board. Participants gave their informed consent voluntarily before gaining access to the survey by ticking a box at the end of a participant information sheet, which included full disclosure and information on study purpose. For underaged participants, their guardian was asked to read the participant information sheet and explain any concepts that their child might not understand (as instructed by the of Finnish National Board on Research Integrity).

The survey consisted of eight demographic questions (e.g., gender, sport discipline, level of proficiency) and scales detailed below. No reward was offered upon completion of the survey. The survey took approximately 15–30 min to complete.

### 2.2 Measures and variables

#### 2.2.1 Emotional abuse

A scale measuring athletes' experiences and observations of emotional abuse by coaches was created for this study. A new scale of 17 items was created as the existing surveys of emotional abuse were deemed insufficient for this study. The new emotional abuse by coaches' survey (EACS) was formed from items of three existing measures of emotional abuse; the Coach-Athlete Relationship Emotional Maltreatment Scale [CAREMS (48), for the prior usage among athletes in the USA, see (49)], the Sport Emotional Response Questionnaire [SER-Q (50), for the prior usage among elite athletes see (51)], and the Controlling Coach Behaviors Scale [CCBS (52), for the prior usage among Icelandic athletes of various disciplines see (53)]. Three items were acquired from each of these surveys. Four items were formed by combining items from both CAREMS and SER-Q. Additionally, three items were added by the authors: “*My coach hits or throws things in front of me *(*e.g.*, *hits the wall*)* when angry, My coach leaves me intentionally outside the team or group (e.g., makes me train on my own)* and *My coach criticizes me (i.e.*, *my appearance, speech or personality)*.” The same items were used for both experienced and observed emotional abuse, however for the latter purpose the items were modified to an observer perspective. For instance, “*Your coach leaves another athlete intentionally outside the team or group.*” Participants were asked to rate their experiences and observations of each item on a 5-point Likert scale from 1 *(Never experienced/observed this*) to 5 (*Always experienced/observed this*).

An exploratory factor analysis was used to test the factor structure of the EACS. The analysis provided a three-factor structure, based on which the following three sum scores were calculated: (1) Verbal abuse (9 items, e.g., “My coach shouts at me in front of others”, the Cronbach's *α* = .0.88), (2) Denial of attention (5 items, e.g., “My coach ignores me if I am ill or injured”, the Cronbach's *α* = .0.78) and (3) Physical abuse [3 items, e.g., “My coach hits or throws things in front of me (e.g., hits the wall) when angry” the Cronbach's *α* = .0.74]. The Kaiser-Meyer-Olkin measure was .95 and Bartlett's test of sphericity was statistically significant. Thus, new factors (from sum scores) were created based on the factor analysis. In addition, a total EACS sum score of 17 items was calculated (Cronbach's *α* = 0.92).

As for the observed emotional abuse (OEACS), an exploratory factor analysis provided a two-factor structure with the Kaiser-Meyer-Olkin measure of .95 and a statistically significant Bartlett's test of sphericity. Based on the factor structure, two sum scores were calculated: (1) Observed denial of attention (6 items, e.g., “Your coach ignores another” athlete if they are ill or injured, the Cronbach's *α* = .0.71) and (2) Observed verbal abuse (11 items, e.g., “Your coach shouts at another athlete in front of you”, the Cronbach's *α* = .0.85). In addition, a total OEACS sum score of 17 items was calculated (the Cronbach's *α* = 0.93).

#### 2.2.2 Athletic identity

The 10-item Athletic Identity Measurement Scale [AIMS (25); for prior usage among international athletes of multiple sports (54)] scale was used as an athletes' athletic identity measure. Participants gave their responses on a 5-point Likert scale ranging from 1 (strongly disagree) to 5 (strongly agree). A confirmatory factor was used to test the four-factor structure suggested by past research [e.g., (55)], and the following sum scores were calculated: (1) Social identity (2 items, e.g., “Most of my friends are athletes”, the Cronbach's *α* = 0.67), (2) Self-identity (2 items, e.g., “I consider myself an athlete”, the Cronbach's *α* = 0.81), (3) Negative affectivity (2 items, e.g., “I feel bad about myself when I do poorly in sport”, the Cronbach's *α* = 0.60), and (4) Exclusivity (3 items, e.g., “Sport is the only important thing in my life”, the Cronbach's *α* = 0.80). Additionally, a total 10-item athletic identity sum score was calculated (the Cronbach's *α* = 0.86).

#### 2.2.3 Disclosure of emotional abuse

Athletes' disclosure of subjectively experienced or observed emotional abuse was measured by one question: “*Did you tell someone about the behavior you experienced or observed?*” Participants were able to choose from the following responses: “*A coach, Teammate or training partner, Another friend, My parent/parents, Another adult, Someone else, I did not tell anyone.*” The item was deemed as the lowest threshold of disclosure of emotionally abusive coaching practices by the study researchers.

#### 2.2.4 Data analysis

Quantitative data analysis was conducted using SPSS version 27. Pearson's correlation analyses were run between the study variables to understand their associations. To compare results between demographic groups One-Way ANOVAs were conducted and where parametric assumptions could not be met, Kruskal-Wallis test was applied. To determine effect sizes, linear regression analyses were carried out between the variables. Assumptions for linear regression were met prior analysis (i.e., normal data distribution, mean distribution error is 0 and error variance is constant). Lastly, a mediation analysis was completed based on the results of the initial correlation and regression analyses.

## 3 Results

### 3.1 Descriptive results

The participants' average rate of experienced emotional abuse was *M* = 1.36 (SD = 0.56), and for observed emotional abuse it was *M* = 1.37(SD = 0.53; Table 2). The participants' average score for athletic identity was *M* = 3.26 (SD = .56). Regarding disclosure of emotional abuse by coaches, 28.8% of the participants had told a parent/guardian. 18.9% told a teammate, 17.3% told a friend, 9% disclosed to another adult and 6.9% had told a coach about the abuse. 19.1% of the participants had not told anyone about the experienced emotional abuse.

**Table 2:** Means (M) and standard deviation (SD) of emotional abuse, athletic identity and disclosure.

Variables	No groups	Gender (*M*) *(SD)*	Age *(M)* (SD)	Level of sport (*M*) (SD)
Experienced emotional abuse	*M* = 1.36, SD = 0.56	Female: 1.37 0.52 Male: 1.29 0.46 Other: 1.80 1.04	12–17: 1.45 0.55 18–30: 1.39 0.53 31–40: 1.25 0.43 41–60: 1.22 0.42 61–80: 1.19 0.35	International: 1.48 0.63 National: 1.41 0.52 Regional: 1.35 0.52 Active: 1.27 0.50 Regular: 1.20 0.39
Observed emotional abuse	*M* = 1.37, SD = 0.56	Female: 1.40 0.57 Male: 1.31 0.52 Other: 1.86 0.80	12–17: 1.43 0.56 18–29: 1.42 0.58 30–39: 1.35 0.63 40–59: 1.25 0.46 60–80: 1.17 0.34	International: 1.57 0.72 National: 1.41 0.54 Regional: 1.38 0.56 Active: 1.30 0.54 Regular: 1.21 0.42
Athletic identity	*M* = 3.26, SD = 0.78	Female: 3.36 0.79 Male: 3.07 0.73 Other: 3.14 0.81	12–17: 3.69 0.65 18–29: 3.38 0.71 30–39: 2.91 0.72 40–59: 2.76 0.66 60–80: 1.17 0.34	International: 3.70 0.64 National: 3.65 0.62 Regional: 3.29 0.68 Active: 3.02 0.69 Regular: 2.57 0.62
Disclosure	*M* = 4.09, SD = 1.83	Female: 3.88 1.72 Male: 3.07 0.73 Other: 3.63 0.77	12–17: 3.20 1.79 18–29: 3.54 2.13 30–39: 4.29 2.17 40–59: 4.01 1.35 60–80: 3.67 2.18	International: 4.25 1.76 National: 4.04 1.66 Regional: 4.10 1.85 Active: 3.69 2.11 Regular: 3.47 1.94

It appeared that the participants observed slightly more emotional abuse, than what they indicated experiencing *χ*^2^(3) = 485.45, *p* < .001. Both female participants and those identifying as other genders reported more instances of experienced *χ*^2^(3) = 23.58, *p* < .001 and observed emotional abuse *χ*^2^(3) = 23.33, *p* < .001, than male participants. However, the other genders had a low representation in the sample, and the result for this group was not significant. The results also revealed that athletes competing at national and international levels reported highest rates of experienced *χ*^2^(6) = 109.82, *p* < .001, and observed emotional abuse *χ*^2^(6) = 118.39, *p* < .001 in comparison to other levels of sports. Additionally, participants within the peak age range for top-level athletes displayed high instances of experienced emotional abuse. Children (aged 12–17) were the group most subjected to witnessing F (4, 1,994) = 8.91, *p* < .001, and experiencing emotional abuse F (4, 1,943) = 1.86, *p* < .001, in comparison to other age groups.

The findings revealed that the participants, overall, exhibited salient levels of athletic identity, with children F (6, 705) = 49.15, *p* < .001, women F (3, 2,505) = 29.05, *p* < .001 and athletes at the international level showing the most salient athletic identities F (6, 2,515) = 181.06, *p* < .001. In terms of disclosure of emotional abuse, athletes generally were willing to report such incidents. International-level athletes, in particular, had the highest disclosure rates (F6, 1,099) = 2.92, *p* < .001. Children disclosed emotional abuse the least F (6, 1,097) = 4.72, *p* < .001. On the other hand, athletes in the 30–39 age bracket were the most disclosing group. Women disclosed the abuse the most in comparison to men and other genders F (3, 1,101) = 19.54, *p* < .001. We did not report the data for equity-deserving groups, as the participation from these communities was low and did not reach statistical significance.

### 3.2 Relationship between athletic identity and emotional abuse

There was a small but significant positive correlation between experienced emotional abuse and athletic identity (*r* = .18, *p* < .001), as well as between observed emotional abuse and athletic identity (*r* = .13, *p* < .001). As shown in Table 3, all dimensions of athletic identity were statistically significantly correlated with all aspects of both experienced and observed emotional abuse.

**Table 3:** Associations of emotional abuse with athletic identity.

Emotional abuse variables	Types of emotional abuse	Social identity	Self-identity	Negative effect	Exclusivity	Disclosure
Experienced emotional abuse	Denial of attention	.15*	.09*	.16*	.14*	−.12*
	Verbal abuse	.17*	.07*	.12*	.25*	−.09*
	Physical abuse	.18*	.08*	.10*	.13*	−.06*
Observed emotional abuse	Denial of attention	.16*	.07*	.12*	.12*	−.12*
	Verbal abuse	.13*	.04*	.10*	.08*	−.11*

* Correlation is significant at the level of *p* < .001.

The results of the linear regression analysis further indicated that that athletic identity was a significant predictor of experienced emotional abuse [F (1, 2,011) = 66.34, *p* < .001, *B* = 0.18, *p* < .001]; the model explained 3.1% of the variance of the experienced emotional abuse. Athletic identity was also a significant predictor of the observed emotional abuse [F (1, 1,960) = 34.49, *p* < .001, *B* = 0.13, *p* < .001]; the model explained 1.7% of the variance. In other words, athletic identity influenced experienced and observed emotional abuse, by increasing the prevalence of the abuse.

### 3.3 Relationship between athletic identity, emotional abuse and disclosure

A low but significant negative correlation was found between experienced emotional abuse and disclosure (of emotional abuse by coaches) (*r* = −.10, *p* < .001), and between observed emotional abuse by coaches and disclosure (*r* = −.08, *p* < .002). As shown in Table 3, the sub-dimensions of both experienced and observed emotional abuse were positively related to the sub-dimensions of athletic identity, but negatively to disclosure.

**Figure 1:**
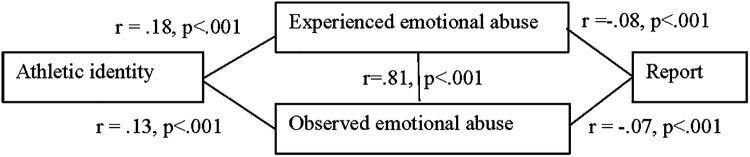
Correlations between athletic identity, observed and experienced emotional abuse, and disclosure of emotional abusive coaching.

The results of a linear regression analysis, summarized in Figure 1, indicated that experienced emotional abuse explained 10% of the variance and that it was a significant predictor of disclosure F (1, 1,959) = 11.18 *p* < .001, *B* = −.07, *p* < .001. A linear regression between observed emotional abuse and disclosure indicated that the abuse explained 0.9% of the variance and that the model was a significant predictor of disclosure F (1, 1,042) = 15.74, B = −.07, *p* < .002. Experienced and observed emotional abuse significantly influenced disclosure, the more emotional abuse athletes experienced and observed, the less likely they were to disclose it.

No significant correlations or regressions were found between athletic identity and disclosure. As seen in Figure 1 athletic identity still had an indirect effect on disclosure through emotional abuse. However, analysis of the sub-dimensions of athletic identity revealed a significant negative correlation between self-identity and disclosure *r* = −0.30, *p* < .02. Self-identity predicted disclosure by reducing the likelihood of disclosure rates.

**Figure 2:**
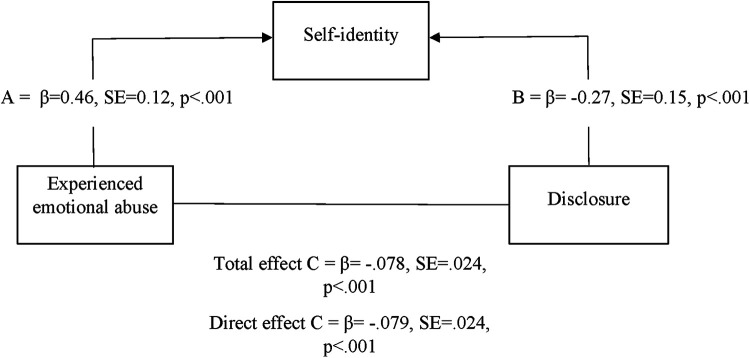
Mediation analysis of self-identity, experienced emotional abuse and disclosure.

A mediation analysis was conducted to explore whether the sub-dimension athletic identity, self-identity, had an influence on the relationship between experienced emotional abuse and athletic identity. In other words, we wanted to explore the mediation effect of self-identity on the discussed relationship. The results in Figure 2 showed that a significant total effect existed between experienced emotional abuse, and disclosure. Direct effects between variables were significant. A Sobel test was conducted to measure the indirect effect *z* = −3.63, SE = 0.03, *p* = .001. The indirect point effect was 0.12. It was concluded that a partial mediation occurred between experienced emotional abuse and disclosure via self-identity. Mediation analysis was not conducted between self-identity, observed emotional abuse and disclosure because the total effect was non-significant.

## 4 Discussion

The study's aim was to ascertain correlations between experienced and observed emotional abuse, athletic identity, and disclosure of emotionally abusive coaching behaviors. The current study is the first to our knowledge to present empirical data on these relationships. First, the initial study results revealed that athletes reported fewer instances and observations of emotional abuse compared to the findings of prior studies [e.g., (56)]. However, it is important to acknowledge that, in the current study, emotional abuse was evaluated using a continuous scale, which might influence the reporting outcomes. Furthermore, participants were found to exhibit salient athletic identities. In contrast to initial expectations, the amount of disclosure of emotional abuse was greater than anticipated, given past research indicating athletes' reluctance to disclose instances of abuse (37).

Second, in-between group research results reaffirmed prior findings that elite athletes experience and observe the most emotional abuse from coaches in comparison to the other levels of sport participation [e.g., (57)]. Female and other gender (e.g., non-binary) athletes reported more emotional abuse than men. While research shows women to be more at risk of sexual abuse and harassment and men of physical abuse [e.g., (9)], emotional abuse shows varying results or no clear gender difference (18, 58). Recent research highlights the vulnerability of sexual and gender minorities to abusive coaching (11, 58). However, the present study suggests that women might be more vulnerable to emotional abuse by coaches.

Moreover, the study unveiled that children were notably susceptible to emotional abuse from coaches when contrasted with other age demographics. This finding aligns with broader research on child abuse within familial and educational settings [e.g., (47)], further emphasizing the vulnerability of children. Children were also the least likely to disclose emotional abuse. In contrast, adults aged 30–39, typically aligning with post-retirement from elite sports, were more likely to disclose emotional abuse. This may be attributed to a diminished athletic identity and a broader sense of self that develops after leaving the competitive sports environment, which could contribute to an increased willingness to discuss abusive experiences (24). In contrast to adults who showed low athletic identities, children had the most salient athletic identities. The findings call attention to the need for research to focus on children within sports contexts and emphasize the importance of educating young athletes about emotional abuse.

Third, our research indicated that a salient athletic identity was associated with experienced emotional abuse by coach. Thus, a salient athletic identity could serve as a risk factor, exposing athletes to emotionally abusive coaching practices. Previous studies indicate that coaches' emotionally abusive actions could be driven by expressive issues, such as poor communication and social skills and instrumental reasons aiming to improve athlete performance (60). These behaviors are prevalent in competitive sports where athletes prioritize success (61, 62). In line, numerous studies indicate that sports culture tends to prioritize athletic performance and success over athletes' physical and psychological wellbeing (18, 63). This culture is embraced by coaches, athletes, and most importantly sport organizations on an international scale (13, 36). The danger with the culture of “winning at all costs” is that it can be used to justify abusive coaching practices, as they may be argued as necessary means for achieving success (13, 64).

Fourth, the results revealed that salient athletic identity was associated with observed emotional abuse. Consequently, emotional abuse is often experienced by more than one athlete at a time and in public spaces with bystanders—individuals not directly involved in either perpetrating or receiving the abuse but who possess the capacity to intervene in these situations—contributing to the public shaming and humiliation of the survivor (5, 65). For instance, coaches have been seen demeaning athletes about their physique in front of others as a misguided form of motivation (21). While it is important for athletes to disclose abusive behaviors, many are deterred by the fear of backlash and pressure to conform to sporting norms (46). Promoting bystander intervention becomes essential in creating a safe environment in sports as it increases the likelihood of investigating and sanctioning perpetrators of abuse (45, 46).

Lastly, the findings showed that both salient athletic identity and emotional abuse negatively predicted athletes' disclosure of emotionally abusive coaching practices. The results suggest that a salient athletic identity decreases the likelihood of disclosure of emotional abuse. More precisely, the results of the mediation analysis revealed that the aspect of athletes' athletic identity influencing disclosure of emotional abuse by coaches was self-identity. Self-identity refers to an athlete's sense of self (value and worth) in the athlete role (25, 26). An athlete with a strong self-identity is likely to interpret situations in terms of their impact on their athletic performance, as most athletes judge their value, self-worth and self-esteem through their role of an athlete (25, 36). In this sense, athletes with a strong athletic identity, and particularly self-identity, who are deeply committed to their athletic success and team affiliation, may overlook the harmful aspects of emotional abuse by a coach if they perceive the abuse as means to achieve their performance goals in sport and as a necessary part of the athlete experience. This interpretation gains further support from previous findings of sport ethic norms. Studies indicate that athletes who exhibit strong adherence to the sports ethic are more likely to experience abuse within the athletic context (23).

### 4.1 Strengths, limitations and future directions

The novelty of the present study lies in that it is the first research that offers insights into the relationships between emotional abuse, athletic identity and disclosure of emotional abuse. The study explored experienced and observed emotional abuse and included children in the participant group. Both aspects are rarely included in abuse research in sports (33). Furthermore, the data sample size is strong for sport psychology research, particularly from a small population country like Finland.

However, the study's broad age range of participants posed challenges in identifying whether the older individuals were active or retired athletes.

Regarding statistical analyses, the regression analyses the models explained only a small percentage of variance (0%–2%). While regression analyses with low R-squared values are acceptable, it indicates that the regression model scarcely fits our data. This could be due to greater unexplainable variation in the data and perhaps other factors affecting the associations between emotional abuse, athletic identity and disclosure. Factors influencing this include individual characteristics like limited sociability and mood states (40).

It should be noted that the reason why emotional abuse, athletic identity and disclosure are associated remains speculative, as the cross-sectional data does not definitively establish causality. Future research should address the questions of how and why the study variables are related by using qualitative research. Furthermore, we want to acknowledge that while this study concentrated on emotional abuse within the athlete-coach relationship abuse also occurs within other relationships (12, 57). Future research should investigate abuse in various sports relationships beyond the coach-athlete dynamic. The insights of coaches on this subject are valuable as they are directly affected, yet often overlooked, in research that predominantly focuses on athletes' experiences (13). Similarly, the perspectives of children and equity-deserving groups are underrepresented in sports research, and their viewpoints are critical to a comprehensive understanding of emotional abuse in sports (1, 33). Diversifying research to encompass these different perspectives is essential (57).

### 4.2 Practical implications

Based on this study's results, future initiatives aimed at preventing and reducing abuse in sports should hence foward acknowledge the role of athletic identity in their safeguarding processes. Athletes must be able to disclose abuse without fear of repercussions, highlighting the need for a safe and empowering environment (40, 66). In this respect, encouraging athletes to develop well-rounded identities that encompass of various life areas, not solely their sport, is key. While sports can still be part of their identity, it should not completely define them. Consequently, a holistic approach to athlete development has been associated with improved performance and increased enjoyment and motivation in athletes [e.g., (27)]. Athletes are unfortunately often discouraged from expanding their identities, due to concerns that anything but a salient athletic identity development may detract athletes from success (27). Coaches in particular often view pursuits outside sports as distractions and may consequently limit athletes' involvement with friends and family (67). This is particularly significant for children, who are more susceptible to the pressures exerted by adults and authority figures to conform to sport ethic norms (23, 35). It is imperative, therefore, that coaches, support staff, and sports organizations facilitate and support the holistic development of athletes' identities (26). To this end, avenues of education such as workshops, seminars, and curated educational materials serve as indispensable tools.

Furthermore, sports organizations must cultivate a culture of trust and accountability surrounding reporting channels. As demonstrated by the results, coming forward with experiences or concerns of abuse is not a straightforward process for athletes. To encourage athletes to report their experiences and observations of abuse it is vital that sport organizations foster a safe environment. Athletes should feel empowered to come forward with their concerns, knowing that their reports will be taken seriously and addressed promptly. To this end researchers have for long advocated for independent reporting, investigation and sanctioning channels for athletes (29, 37). Establishing clear communication channels and upholding a transparent approach during the reporting procedure is crucial (66). Additionally, survivors should be consistently kept informed and provided with timely updates about their case, providing the survivors with a sense of control and assurance (37, 66). Survivors should also be provided with opportunities for delivering feedback and dialogue about the disclosure process, ensuring any concerns or questions they have are adequately addressed and their voices heard throughout the process (37, 66).

## 5 Conclusion

This research extends the growing body of work examining factors that contribute to emotional abuse in the athlete-coach relationship. This study aimed to show the intricate and challenging nature of emotional abuse within sports and to illustrate the harmful nature of a salient athletic identity. In summary, the study findings highlight salient athletic identity as a risk factor that can expose athletes to emotional abuse by coaches, both experienced and observed. We particularly want to emphasize the vulnerability of children to emotional abuse by coaches. In this study, athletic identity is also presented as a barrier that prevents disclosure of emotionally abusive coaching practices. Ultimately, by recognizing and addressing the complex interplay between athletic identity, emotional abuse, and disclosure of this abuse in sports, we can work towards creating safer and more inclusive sporting where children's and human rights are upheld, and athletes feel empowered to speak out against abuse without fear of repercussions. The responsibility of this lies with all adults in the sport sector.

## Data Availability

The presented dataset in this article is not readily available because all members of the research group need to accept any requests to data. Access can be provided upon request and approval by the researchers. Requests to access the datasets should be directed to jatta.muhonen@helsinki.fi.
